# m^6^A demethylase–driven reprogramming of leukemia-associated macrophages predicts improved outcomes in acute myeloid leukemia

**DOI:** 10.3389/fimmu.2026.1739959

**Published:** 2026-02-03

**Authors:** Zhiyu Shi, Yuan Xia, Mingyue Zhang, Ying Peng, Yun An, Qingjun Zhu, Tao Sun

**Affiliations:** 1 Innovation Research Institute of Traditional Chinese Medicine, Shandong University of Traditional Chinese Medicine, Jinan, China; 2 Department of Hematology, Qilu Hospital of Shandong University, Cheeloo College of Medicine, Shandong University, Jinan, China; 3 School of Rehabilitation Medicine, Shandong University of Traditional Chinese Medicine, Jinan, China; 4 Key Laboratory of Traditional Chinese Medicine Classical Theory, Ministry of Education, Shandong University of Traditional Chinese Medicine, Jinan, China

**Keywords:** acute myeloid leukemia, immune microenvironment, macrophage, N6-methyladenosine modification, single-cell RNA-seq

## Abstract

**Background:**

N6-methyladenosine (m^6^A) is a dynamic mRNA modification influencing transcript fate and cellular identity, especially in cancer. While oncogenic roles of m^6^A regulators in AML cells are known, their impact on the leukemic immune microenvironment is unclear.

**Methods:**

In this study, we constructed a single-cell atlas of macrophages in AML by integrating publicly available scRNA-seq datasets from 129 patient cohorts. Data were batch-corrected using Seurat and Harmony. Macrophage subpopulations were identified, and the expression and activity of 29 m^6^A regulators were analyzed. Pseudotime analysis (Monocle3), cell–cell communication (CellChat), and pathway enrichment (Metascape) analyses were performed to explore m^6^A-related functional programs. Survival analysis was conducted using Kaplan–Meier curves. RT-qPCR was used to verify the correlation between m^6^A regulatory molecules and prognosis.

**Results:**

Our findings indicated that m^6^A regulators are associated with macrophage fate. Writer-high macrophages showed enhanced proliferation and differentiation, maintaining monocyte-like features. Eraser-high macrophages remodeled macrophage function toward an M1-like, pro-inflammatory and antigen-presenting state. Reader-high macrophages drove macrophages toward an immunosuppressive, M2-like phenotype, while m^6^A-deficient cells exhibit features of functional exhaustion. Survival analysis based on bulk RNA-seq data further revealed that m^6^A-regulated macrophage profiles were associated with distinct prognostic stratification in AML patients. RT-qPCR analysis of macrophages isolated from clinical AML samples further validated these findings, showing that patients with favorable prognosis exhibited significantly higher expression levels of erasers compared to those with poor prognosis.

**Conclusion:**

These results highlight m^6^A system’s role in macrophage reprogramming and suggest that targeting m^6^A regulators in macrophages may serve as a potential basis for prognostic stratification and a promising therapeutic strategy in AML.

## Introduction

Acute myeloid leukemia (AML) is a heterogeneous hematologic malignancy characterized by the clonal expansion of myeloid precursors with impaired differentiation (1). Although significant progress has been made in genomics and immunophenotyping, the tumor microenvironment (TME), especially the role of immune cells in leukemogenesis and disease progression, remains poorly understood. N6-methyladenosine (m^6^A), the most abundant internal modification in eukaryotic mRNA, is among the many epigenetic regulators implicated in AML. Recent studies have demonstrated that dysregulation of m^6^A modification plays a significant role in AML development. Many m^6^A regulators such as METTL3 (2), FTO (3), and YTHDF2 (4) have been shown to promote AML cell proliferation and survival. Nevertheless, the impact of m^6^A modification on immune regulation within the TME, especially in innate immune cells like macrophages, remains largely elusive.

Tumor-associated macrophages (TAMs) display considerable phenotypic plasticity. They can be reprogrammed from a pro-inflammatory M1-like state to an immunosuppressive M2-like and exhaustion phenotype (5), facilitating immune evasion, reducing therapeutic responsiveness (6), and ultimately driving disease progression and relapse (7). m^6^A modification is closely associated with macrophage phenotypes and functional dysregulation. Distinct macrophage phenotypes exhibit markedly different m^6^A modification patterns. Previous studies in solid tumor have demonstrated that METTL3 (8) and YTHDF1 (9) promote M1 polarization of macrophages, whereas IGF2BP2 (10) and YTHDF2 (11) facilitate M2 polarization. Functionally, macrophage activation is regulated by METTL3 (12), while ALKBH5 (13) has been shown to modulate macrophage senescence, and HNRNPC (14) plays a role in the production of pro-inflammatory cytokines by macrophages. We hypothesize that m^6^A also regulates leukemia-associated macrophages (LAMs) function in AML, shaping the immune microenvironment. Yet, most studies have examined only individual regulators, leaving the integrated system poorly understood. Here, we provide a comprehensive analysis of multiple m^6^A regulators to reveal their coordinated roles in macrophage function and fate.

In this study, we systematically analyzed publicly available single-cell RNA sequencing (scRNA-seq) datasets encompassing AML patient samples, constructed atlas of AML-derived macrophages. By integrating expression signatures of m^6^A writers, erasers, and readers with canonical macrophage gene sets, we identified four m^6^A-regulated subsets that are specifically associated with distinct activation states of macrophages. Our analysis reveal that m^6^A regulators coordinate multiple reprogramming processes, regulating macrophage polarization and functional transformation. Our findings uncover the molecular mechanisms of m^6^A dysregulation in LAMs and demonstrate how distinct regulators affect their reprogramming, leading to divergent effects on AML prognosis. These results highlight the impact of epitranscriptomic regulation on the AML immune microenvironment and, by defining the role of m^6^A in macrophage biology, provide new insights into the epigenetic–immune interface and reveal and reveal potential prognostic and therapeutic targets.

## Materials and methods

### Quantification of m^6^A regulator expression and activity

We obtained 29 m^6^A regulators, which including 11”writers” (METTL3, METTL14, METTL16, WTAP, METTL5, ZC3H13, CBLL1, RBM15, RBM15B, PCIF1, ZCCHC4),3 “erasers”(ALKBH5, FTO, ALKBH3) and 15 “readers”(IGF2BP3, IGF2BP2, YTHDC1, YTHDC2, YTHDF1, YTHDF2, YTHDF3, HNRNPA2B1, HNRNPC, HNRNPH1, LRPPRC, ELAVL1, FMR1, RBM33, RBMX) (15–21). m^6^A modification activity was scored using the AUCell R package, which evaluates the enrichment of predefined gene sets at the single-sample level based on the area under the recovery curve of gene expression rankings. To assess the robustness of the scoring strategy, we additionally applied the singscoreR package (22), an independent rank-based method that calculates normalized scores by comparing the relative expression ranks of signature genes within each sample, thereby minimizing the influence of between-sample normalization and technical variability.

### Trajectory and pseudotime analysis

To explore macrophage differentiation dynamics, pseudotime trajectories were constructed using Monocle3 (v1.2.9). Pseudotime trajectory analysis was performed using Monocle3. The Seurat object was first converted into a cell_data_set. Data were preprocessed using preprocess_cds with principal component analysis (num_dim = 50) to capture major transcriptional variation. Cells were clustered using the Leiden algorithm implemented in cluster_cells (k = 20, resolution = 1e−3). Trajectory inference was conducted using learn_graph with default settings (use_partition = TRUE, close_loop = FALSE) to learn a principal graph representing transcriptional state transitions. Pseudotime ordering was performed using order_cells, with the root defined as the Mono.M cluster, which represents the biologically earliest cell state based on lineage marker expression and relative pseudotime distribution.

### Data collection and preprocessing

Publicly available single-cell RNA sequencing (scRNA-seq) datasets from patients with acute myeloid leukemia (AML) were retrieved from the Gene Expression Omnibus (GEO), ArrayExpress, and published supplementary sources. Inclusion criteria were (1): availability of raw or preprocessed gene expression matrices (2), annotation or identifiable markers for immune cells, and (3) inclusion of bone marrow or peripheral blood mononuclear cells from AML patients. A total of 10 datasets encompassing n = 129 AML patients were included (**Supplementary Table S1**).Raw count matrices were processed using the Seurat package (v4.3.0) in R. Cells with fewer than 500 detected genes, >20% mitochondrial gene content, or doublet-like profiles were excluded. Genes expressed in fewer than 5 cells were filtered out. Datasets were normalized using Harmony to correct for batch effects.

### Immune cell and macrophage annotation

Cell clustering was performed using principal component analysis (PCA), followed by uniform manifold approximation and projection (UMAP) for dimensionality reduction. Clusters were identified using a shared nearest neighbor (SNN) graph–based clustering approach with a resolution of 0.5, followed by marker-based cell type assignment (23, 24). Immune cell subsets were annotated based on canonical markers (25–27). Macrophage-lineage cells were subsetted for downstream analysis.

### Differential expression and gene set enrichment analysis

Differential gene expression (DGE) analysis was performed using FindMarkers in Seurat with Wilcoxon rank-sum test. FindAllMarkers performs pairwise comparisons of each cluster against all other cells to identify genes that are significantly up- or down-regulated. Adjusted p-values < 0.05 and |log2FC| > 0.25 were considered significant. To identify the functions of DEGs in AML subsets. Gene Ontology (GO) was conducted using the Metascape (https://metascape.org/) The results of enrichment analysis are shown using corrected p values and normalized enrichment scores.

### Cell-cell communication analysis

Intercellular communication in AML cells and immune cells was analyzed using the CellChat package. Expression matrices were extracted from the Seurat object, and ligand-receptor interactions were identified using the CellChatDB.human database. Communication probabilities (computeCommunProb) and pathway-level communication strengths (computeCommunProbPathway) were calculated, and intercellular communication networks were aggregated (aggregateNet). Differences between tumor and normal tissues were compared by signaling pathway activity (netAnalysis_computeCentrality) and visualized via network diagrams (netVisual_diffInteraction) and heatmaps (netVisual_heatmap).

### PPI network construction

The upregulated DEGs and m^6^A regulators were used to explore their interactions using the Search Tool for the Retrieval of Interacting Genes/Proteins (STRING) online database (https://string-db.org/), with a median confidence score cutoff of 0.4. The protein-protein interaction (PPI) network was visualized and analyzed using Cytoscape (version 3.9.0).

### Survival analysis

Overall survival (OS) was defined as the time from diagnosis to death or last follow-up. Differences in OS among subtypes within each cohort were assessed using Mantel-Cox log-rank tests implemented in the R package survival. Kaplan–Meier curves for each cluster were visualized with the survminer package. To evaluate the predictive performance of the risk model, 1-, 3-, and 5-year receiver operating characteristic (ROC) curves were generated using the survivalROC package (v1.0.3.1). Associations analyzed using Cox proportional hazards regression implemented in the survival R package. Hazard ratios with 95% confidence intervals were used to summarize the associations, and the results were visualized using ggplot2.

### Clinical sample collection

Bone marrow samples from AML patients were collected at Qilu Hospital of Shandong University, with informed consent obtained and ethical approval granted. All the patients have signed the informed consents.

### RT-qPCR

Total RNA was extracted using TRIzol reagent (Thermo Fisher Scientific, #15596026) following the manufacturer’s instructions. Complementary DNA (cDNA) was synthesized from 500 ng RNA using the PrimeScript™ RT Master Mix (Takara, Japan, #RR036A) in a 20 µL reaction under the following conditions: 37°C for 15 min, followed by 85°C for 5 s to inactivate the reverse transcriptase. Quantitative real-time PCR (qRT-PCR) was performed with TB Green Premix Ex Taq™ II (Takara, #RR820A) on a LightCycler 480 II system (Roche) using the following cycling program: 95°C for 30 s, followed by 40 cycles of 95°C for 5 s and 60°C for 30 s. Melt curve analysis was performed to confirm specificity of amplification. GAPDH was used as an endogenous control. Primer sequences for all genes analyzed are listed in **Supplementary Table 3**.

### Flow cytometric sorting of macrophages

Mononuclear cells were isolated from bone marrow aspirates of AML patients by Ficoll-Paque density gradient centrifugation. After washing with PBS containing 2% fetal bovine serum (FBS), cells were incubated with human Fc receptor blocking reagent (Miltenyi Biotec) to minimize nonspecific binding, followed by staining with fluorochrome-conjugated antibodies against CD11b and CD68. Macrophages were defined as CD11b^+^CD68^+^ cells and sorted using a BD FACSAria™ III cell sorter. The purity of the sorted macrophage population was routinely greater than 95%, as confirmed by post-sort analysis.

### Statistics

All experiments were performed with a sample size of at least three biological replicates (n ≥ 3). Data are presented as the mean ± standard deviation (SD). Differences between two groups were evaluated using an unpaired t-test or correlation analysis, as appropriate. Two-way ANOVA was applied to assess the main and interactive effects of two factors between groups. A P value < 0.05 was considered statistically significant. Statistical analyses were performed using GraphPad Prism 9.0 or the R statistical environment.

## Results

### Different classes of m^6^A regulators are associated with distinct macrophage phenotypes

**Figure 1:**
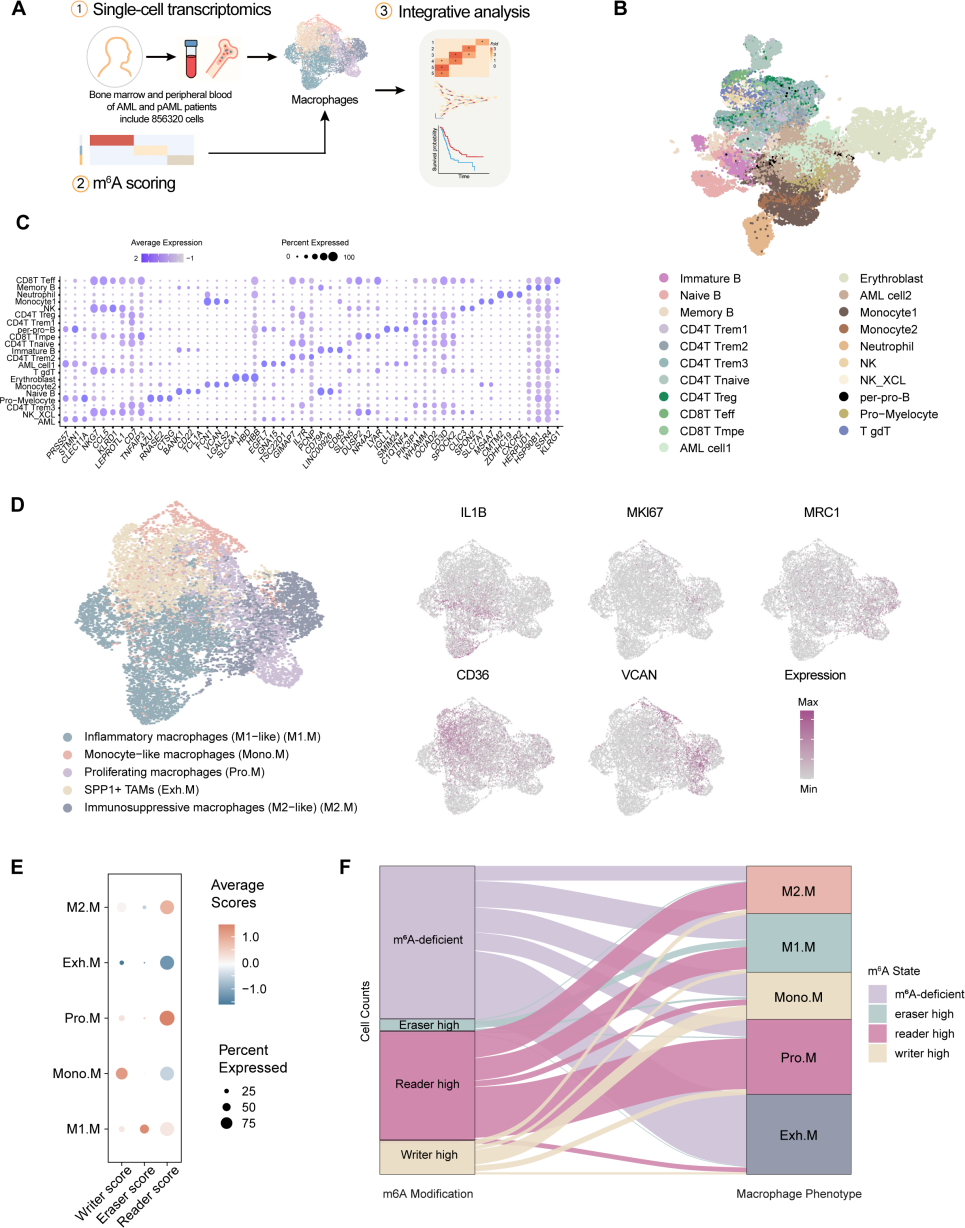
Different classes of m^6^A regulators are associated with distinct macrophage phenotypes. **(A)** Schematic overview of the analysis workflow. Single-cell RNA-seq data from bone marrow and peripheral blood of AML patients were reanalyzed. Macrophages were extracted for integrative analysis of m^6^A regulator expression. **(B)** Uniform manifold approximation and projection (UMAP) plot of annotated cell populations from AML samples, showing major immune and hematopoietic lineages. **(C)** Dot plot showing the expression of representative marker genes across identified immune cell types. Dot size indicates the proportion of expressing cells; color indicates scaled average expression. **(D)** UMAP plots of macrophages highlighting subclusters and representative markers (IL1B, MKI67, MRC1, CD36, VCAN). **(E)** Dot plot displaying average expression levels of m^6^A writer, eraser, and reader genes across macrophage subtypes.**(F)** Sankey diagram showing the correspondence between m^6^A modification states (writer-high, reader-high, eraser-high, or m^6^A-deficient) and macrophage phenotypes.

To investigate the role of different kinds of m^6^A regulators in shaping macrophage states within the AML microenvironment, we reanalyzed publicly available scRNA-seq datasets (**Supplementary Table 1**), comprising a total of 856,320 cells derived from the bone marrow and peripheral blood of AML patients (**Figure 1A**; **Supplementary Figure 1A**). Based on distinct lineage-specific marker gene sets, the overall cellular landscape of AML patients was delineated, confirming the presence of diverse hematopoietic and immune lineages (**Figures 1B, C**). To further validate our cell type annotations, we extracted the original annotations from the GSE116256 dataset and systematically compared them with our own classifications. In addition, we examined the marker genes associated with the original clusters and compared them with those defining our clusters, demonstrating a high degree of consistency between our annotations and the previously published classifications (**Supplementary Figures 1B, C**). Through this validation, a total of 140,166 macrophages were identified and confirmed to represent normal immune cells rather than malignant cells (25–27), which were subsequently included in downstream stratification analyses (**Figure 1D**). This revealed five transcriptionally distinct macrophage phenotypes: monocyte-like macrophages, inflammatory (M1-like) macrophages, proliferating macrophages, immunosuppressive M2-like macrophages, and a unique population of SPP1^+^ LAMs characterized by high expression of exhaustion and remodeling markers.

To systematically assess m^6^A modification states, we curated a panel of 29 well-characterized regulators (**Supplementary Table 2**), comprising three classes (writers, erasers and readers), and applied AUCell to score their expression within individual cells. The resulting area under the ROC curve (AUC) reflects the discriminative ability of each score, with higher values indicating better predictive performance. m^6^A scoring uncovered distinct modification patterns across macrophage subsets (**Figure 1E**). To further validate the robustness of the m^6^A scoring strategy, we employed singscore as an independent scoring method (22). Singscore-based evaluation of m^6^A writers, erasers, and readers yielded highly consistent results with our original m^6^A score (**Supplementary Figure 1E**). Monocyte-derived macrophages were prominently marked by elevated expression of writer genes, while M1-like macrophages were enriched for eraser activity. In contrast, both M2-like and proliferative macrophages exhibited high reader scores. Notably, the SPP1^+^ LAMs exhibited minimal scores of m^6^A system across all three categories, suggesting uniformly low scores of m^6^A regulators, potentially associated with functional exhaustion. The cell proportion analysis further substantiated these relationships, underscoring the specific associations between the three m^6^A regulator states and distinct macrophage phenotypes (**Figure 1F**), suggesting that m^6^A modification may potentially regulate macrophage plasticity in AML.

### Four subsets exhibited potential pathway features associated with distinct macrophage phenotypes

**Figure 2:**
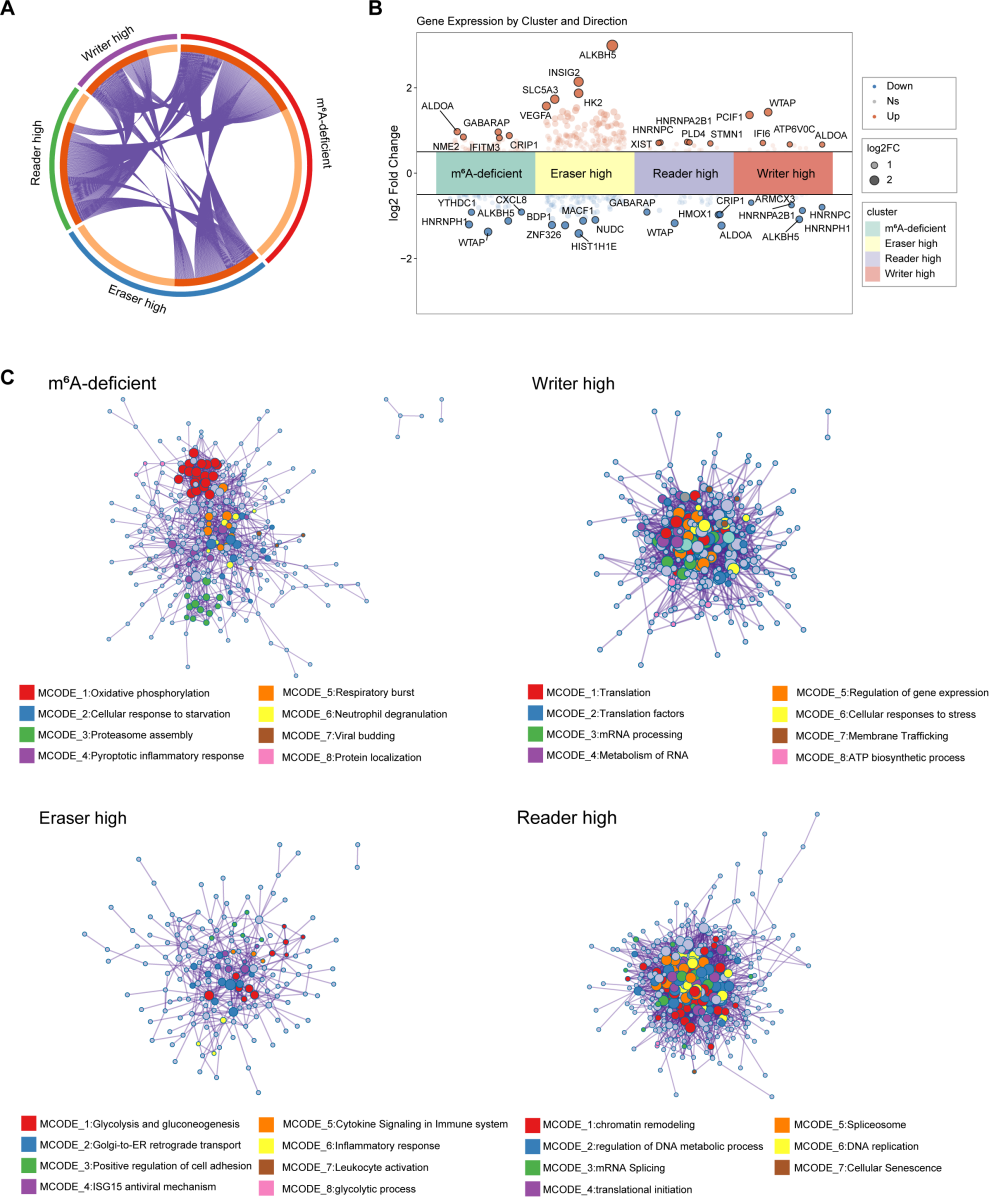
Four subsets exhibited potential pathway features associated with distinct macrophage phenotypes. **(A)** Chord diagram showing overlap of differentially expressed genes between macrophage subsets grouped by m^6^A state (m^6^A-deficient, writer_high, eraser_high, reader_high). **(B)** Selected differentially expressed genes across subsets, highlighting both functional genes and m^6^A regulators. Abbreviations: NS, not significant; logFC, log fold change. **(C)** Functional enrichment networks (MCODE clusters) based on subset-specific upregulated genes. Enriched GO terms are grouped by functional modules; node size reflects gene count; colors denote pathway categories.

We focused our analysis on macrophages with specific m^6^A scores (writer-high, eraser-high, reader-high and m^6^A-deficient). We compared the molecular and functional profiles of these four m^6^A-regulated macrophage subsets. Differentially expressed genes (DEGs) for each subset were identified using FindAllMarkers function, comparing cells in the subset (target group) against all other macrophages (comparison group).While some Differentially Expressed Genes (DEGs) were shared among the subsets (**Figure 2A**), each exhibited a distinct DEGs and displayed unique expression patterns characteristic of their respective states (**Figures 2A, B**).

We next examined the functional programs enriched in each subset. m^6^A-deficient macrophages exhibited stress-related transcriptional signatures, including hypoxia, nutrient deprivation, and pyroptotic inflammation. These features were consistent with an exhausted or dysfunctional phenotype, characterized by impaired metabolic activity and diminished immune function. Writer-high macrophages were enriched in proliferation-related and translation-related pathways, indicating a highly active functional state that aligned with their association with monocyte-derived, actively cycling macrophages. Eraser-high cells exhibited strong enrichment for pro-inflammatory programs and chemokine signaling, reflecting an M1-like immune-activating profile. Reader-high macrophages showed dominant signatures related to chromatin remodeling, gene silencing, and post-transcriptional control, suggestive of an immunoregulatory or suppressive phenotype (**Figure 2C**; **Supplementary Figures 2A, B**). Together, these findings indicated that distinct m^6^A modification states may influence macrophage biological functions by regulating epigenetic modifications, thereby shaping the fate of macrophages within the AML microenvironment.

### Different classes of m^6^A regulators drive macrophage differentiation

**Figure 3:**
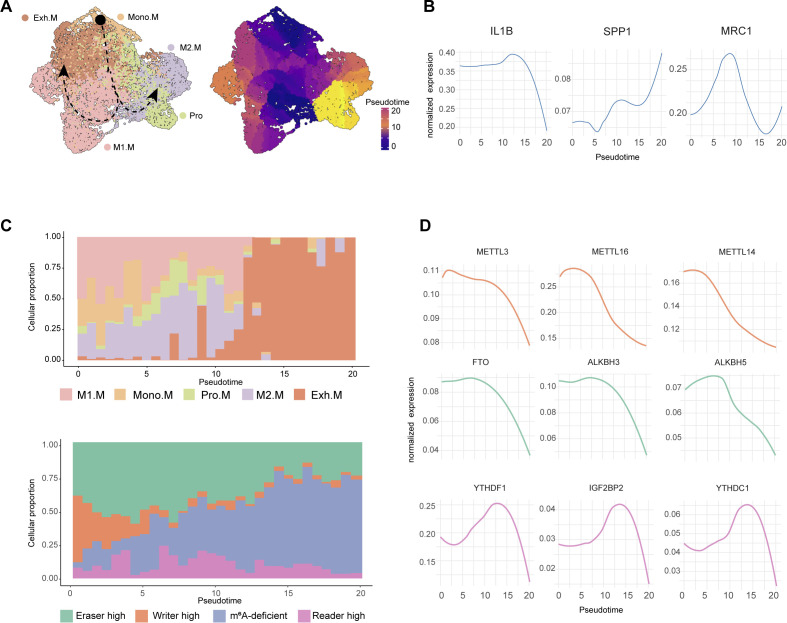
Different classes of m^6^A regulators drive macrophage differentiation. **(A)** Left: UMAP embedding of macrophages annotated by subtype, with arrows indicating developmental paths inferred by Monocle3. Right: same cells colored by pseudotime. **(B)** Expression of canonical macrophage markers (IL1B, SPP1, MRC1) plotted along pseudotime. **(C)** Stacked bar plots showing the proportion of macrophage subtypes (top) and m^6^A modification states (bottom) along pseudotime. **(D)** Expression dynamics of selected m^6^A writers (top row), erasers (middle), and readers (bottom) across pseudotime.

We used Monocle3 to infer pseudotime trajectories and assess the potential role of m^6^A modification in shaping macrophage fate decisions during differentiation. Monocyte-derived macrophages were located at the root of the trajectory, which bifurcated into distinct branches corresponding to inflammatory M1-like, immunosuppressive M2-like, and terminally exhausted macrophage states (**Figure 3A**). Systematic changes in the expression of canonical macrophage markers (IL1B, SPP1, and MRC1) along the pseudotime trajectory further validated the inferred differentiation path and its biological relevance (**Figure 3B**).

Next, we examined the distribution of macrophage subsets in and their corresponding m^6^A modification states along pseudotime. We observed that the early trajectory was dominated by Mono.M cells with high writer activity, followed by a transient expansion of eraser-high macrophages corresponding to M1-like states. Along the differentiation trajectory, reader-high cells, encompassing M2-like and proliferative macrophages, progressively dominated the population. Terminally exhausted macrophages showed a widespread decline in m^6^A-related signals, consistent with an m^6^A-deficient phenotype (**Figure 3C**).

Finally, we evaluated the dynamic expression of individual m^6^A regulators across pseudotime. Most writer (METTL3, METTL14), eraser (FTO, ALKBH5), and reader (YTHDF1, IGF2BP2) genes showed coordinated expression changes along the differentiation trajectory (**Figure 3D**). These findings support the idea that different classes of m^6^A regulators drive macrophage differentiation at distinct stages, writer-high macrophages dominate early development, followed by eraser-high cells driving M1 activation, then reader-high macrophages promoting an M2 phenotype, and finally m^6^A silencing leading to macrophage exhaustion, highlighting distinct roles of m^6^A regulators in macrophage fate.

### Different classes of m^6^A regulators modulate macrophage phenotypes via diverse targets

**Figure 4:**
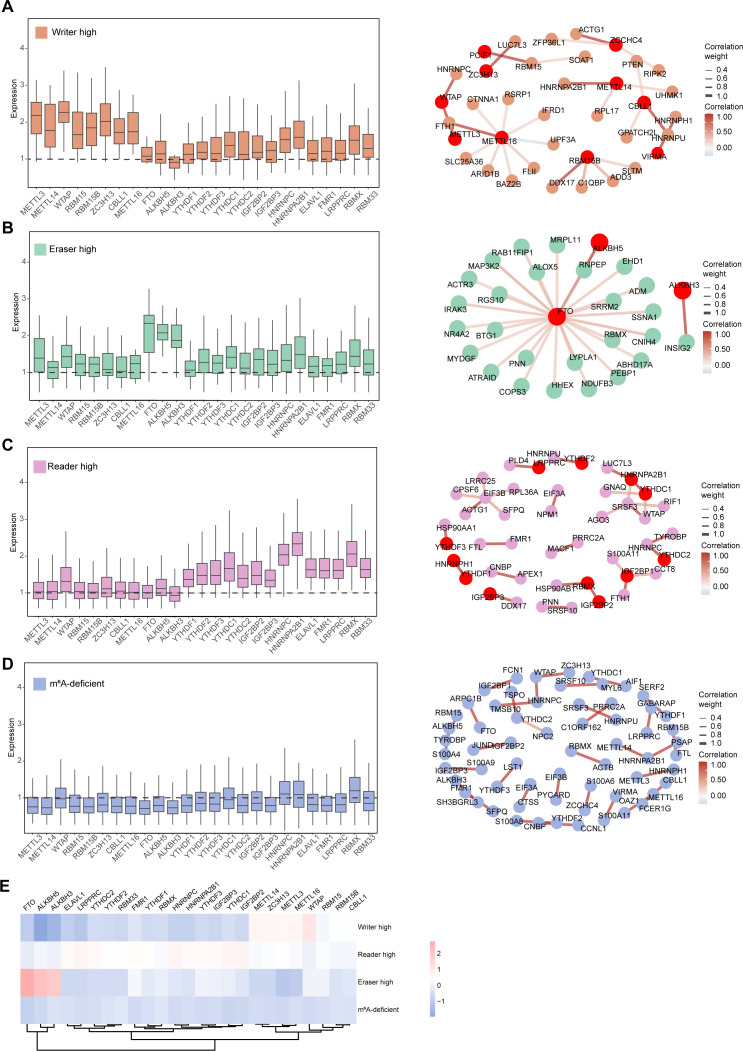
Different classes of m^6^A regulator modulate macrophage phenotypes via diverse targets. **(A–D)** Left: Boxplots showing expression levels of m^6^A-related genes in writer-high **(A)**, eraser-high **(B)**, reader-high **(C)**, and m^6^A-deficient **(D)** macrophage states. Right: Correlation networks constructed between highly expressed m^6^A regulators and their positively associated downstream genes (Pearson’s r > 0.4, p < 0.05). Edge thickness denotes correlation strength, node size indicates average expression, and color represents m^6^A group–specific enrichment. **(E)** Heatmap showing the scaled expression levels of m^6^A writers, readers, and erasers across the indicated m^6^A subsets. Rows represent m^6^A subgroups, and columns denote individual m^6^A regulatory genes. Hierarchical clustering was performed based on gene expression patterns. Color intensity indicates relative expression levels (z-score).

To reveal how different classes of m^6^A regulators orchestrate macrophage state transitions and fate determination, we constructed correlation-based regulatory networks centered on subset-enriched m^6^A regulators and DEGs. In writer-high cells, we observed consistent upregulation of writer genes, including METTL3, METTL14, WTAP, and RBM15 (**Figure 4A**, left). Downstream genes of these categories exhibit dense network connectivity and reflect a highly active, metabolically dynamic state. This state is characterized by elevated transcriptional activity (RPL17, UPF3A, RSRP1), enhanced metabolic processes (SOAT1, SLC25A36, FTH1), and increased proliferative capacity (ACTG1, CTNNA1) (**Figure 4A**, right). These findings indicate that m^6^A writers potentially promote macrophage activation, metabolic activity, and proliferation, contributing to unique phenotypic states in the AML microenvironment.

In eraser-high macrophages (**Figure 4B**), which were primarily associated with inflammatory states, ALKBH5 and FTO exhibited strong positive correlations with genes involved in cytokine production and innate immunity, including IRAK3, ALOX5, and NLRP3. This network architecture corresponded to the inflammatory phenotype characteristic of M1-like macrophages and suggest that m^6^A erasers may be involved in macrophage reprogramming, promoting their transition toward an inflammatory phenotype. Reader-high cells displayed a distinct regulatory landscape (**Figure 4C**), where high expression of YTHDF1, IGF2BP2, and YTHDC1 correlated with genes involved in RNA processing, chromatin remodeling, and immune evasion pathways (PRPF3, RPS6, TRIM28). m^6^A readers may orchestrate a program favoring immune evasion and cellular plasticity through enhanced RNA processing and chromatin remodeling.

m^6^A-deficient macrophages showed uniformly low expression of m^6^A regulators (**Figure 4D**). The gene network extensively connects immune-related genes (TYROBP, FCER1G, S100A8 and S100A9), potentially influencing immune and inflammatory responses that contribute to the development of exhaustion. In addition, metabolic-related genes (FTL and TSPO) are also involved, possibly regulating metabolism and cellular energy status, thereby impacting the exhaustion state. m^6^A-deficient represented a distinct regulatory state characterized by low expression of m^6^A regulators and a chaotic and intricate regulatory landscape, leading to disorganized gene expression and impaired cellular function. In summary, distinct m^6^A regulators drive divergent macrophage states through specific downstream gene networks, writers promote activation and proliferation, erasers support inflammatory programs, readers facilitate immune evasion by modulating RNA processing, and m^6^A-deficient cells exhibit dysregulated networks leading to exhaustion (**Figure 4E**).

### Four m^6^A -regulated subsets communicate through distinct signaling axes

**Figure 5:**
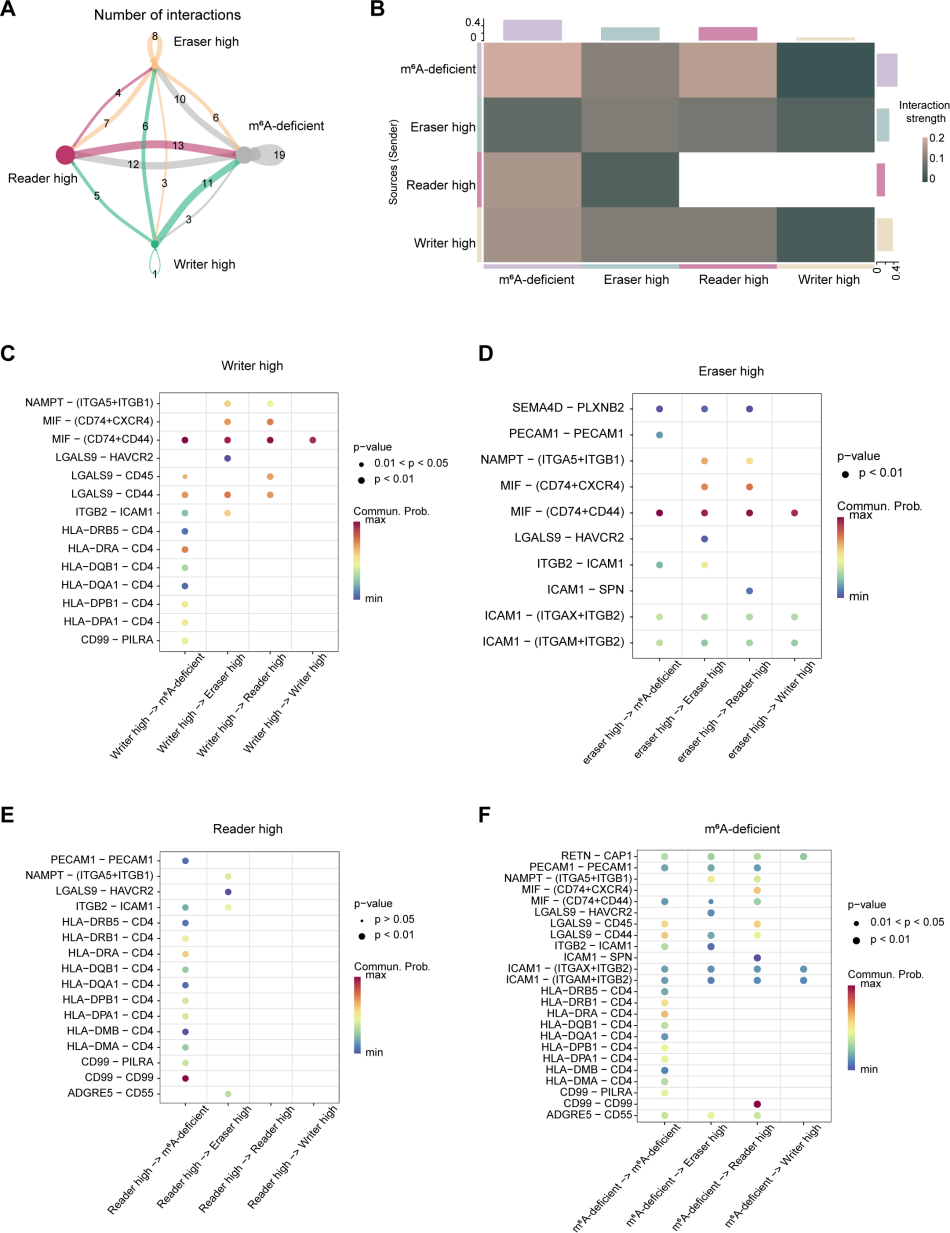
Four m^6^A -regulated subsets communicate through distinct signaling axes. **(A)** Chord diagram summarizing total number of ligand–receptor interactions among four m^6^A-regulated macrophage subtypes. **(B)** Heatmap displaying communication strength (computed using CellPhoneDB or similar tool) between sender and receiver macrophage populations. **(C–F)** Dot plots showing key ligand–receptor interactions originating from writer-high **(C)**, eraser-high **(D)**, reader-high **(E)**, and m^6^A-deficient **(F)** macrophages. Dot size indicates significance (p < 0.05 or 0.01), and color represents communication probability.

We examined the cell–cell communication patterns of macrophages to assess how distinct m^6^A states influence their interactions within the AML microenvironment. Focusing on ligand–receptor networks, our analysis revealed an interaction landscape characterized by marked differences among the four subsets (**Figures 5A, B**). Notably, eraser-high macrophages exhibited reduced outgoing signaling. In contrast, writer-high cells primarily function as signal senders but have limited incoming interactions. Reader-high macrophages showed few detectable connections with other macrophage subsets and mainly interact with m^6^A-deficient cells, suggesting they may exist in an isolated state. Conversely, m^6^A-deficient macrophages maintained extensive outgoing communication, potentially exerting broader influence on other cells and the tumor microenvironment.

Dissecting the ligand-receptor pairs revealed subset-specific signaling axes. Writer-high cells predominantly transmitted MIF-(CD74+CD44) signaling to other macrophage subsets (**Figure 5C**), a known axis for modulating macrophage activation. Interestingly, eraser-high cells also engaged this pathway, underscoring its central role in immune activation and its capacity to reprogram surrounding cells toward a pro-inflammatory and activated state (**Figure 5D**). Reader-high macrophages exhibited limited intercellular interactions, with CD99-CD99 homotypic signaling being the most prominent, particularly directed toward exhausted macrophages (**Figure 5E**). CD99-CD99 interactions are known to promote cell proliferation, which may lead to an expansion of exhausted macrophages and thereby contribute to the formation of an immunosuppressive niche in the AML microenvironment. Similarly, m^6^A-deficient cells exhibited enhanced CD99-CD99 signaling toward reader-high cells, promoting the formation and aggravation of the immunosuppressive microenvironment (**Figure 5F**). Interestingly, m^6^A-deficient macrophages uniquely received HLA-CD related signals from other subsets, a signal typically associated with antigen presentation and adaptive immune activation, potentially indicative of a process aimed at reinvigorating these cells, reversing their exhausted phenotype and reinstating immune functionality.

In addition, we performed cell–cell communication analyses between macrophage subsets and T cells. The results showed that the eraser-high subset was significantly enriched in pro-inflammatory signaling pathways, suggesting a role in inflammatory activation and immune cell recruitment. Cell–cell communication analysis between macrophages and AML cells revealed that eraser-high macrophages predominantly interacted with AML cells through immune-related pathways, indicating an immune-interactive, antigen presentation–associated microenvironment. In contrast, Reader-high and m^6^A-deficient subsets exhibited signaling patterns associated with enhanced leukemic cell proliferation and immune evasion (**Supplementary Figures 3A–D**). These findings suggest that the eraser-high subset may exert its effects by promoting effector T-cell activation, thereby influencing AML progression.

### Eraser-high macrophages are associated with favorable prognosis in AML

**Figure 6:**
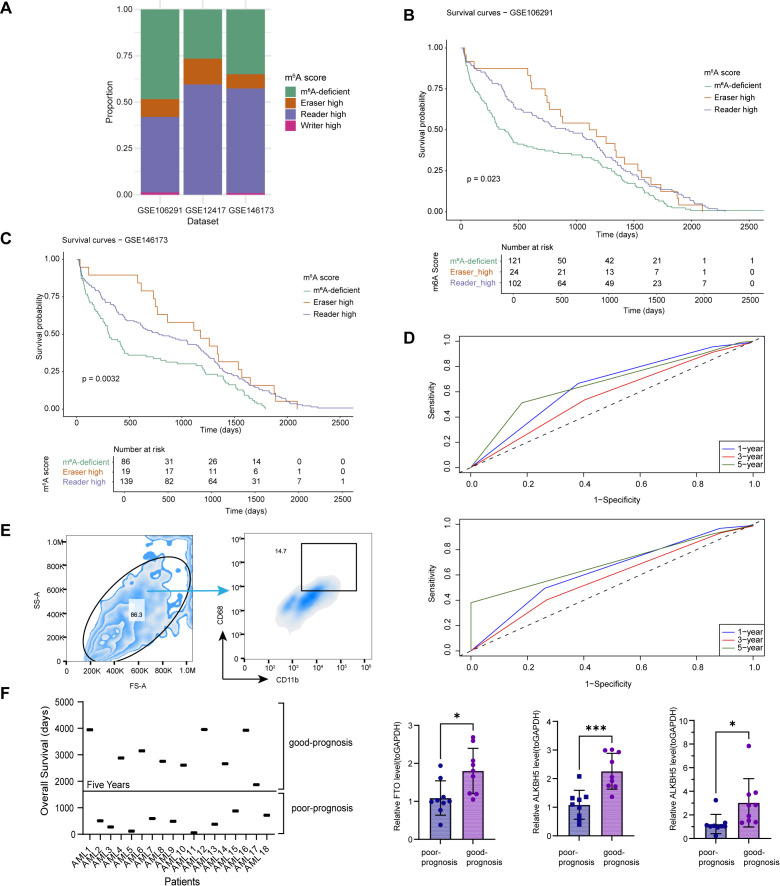
Eraser-high macrophages are associated with favorable prognosis in AML. **(A)** Barplot showing the proportion of four m^6^A-regulated macrophage subtypes across three AML bulk RNA-seq datasets (GSE106291, GSE12417.79, GSE146173). **(B, C)** Kaplan–Meier survival curves for m^6^A macrophage subgroups in GSE106291 **(B)** and GSE146173 **(C)**. The writer-high group was excluded due to low sample count. **(D)** Time-dependent ROC curves evaluating 1-, 3-, and 5- year survival prediction performance of the m^6^A macrophage signature in two datasets. **(E)** Mononuclear cells isolated from AML patient bone marrow were first gated based on forward scatter (FSC-A) and side scatter (SSC-A) to exclude debris and doublets. CD11b^+^CD68^+^ cells were subsequently identified and sorted as macrophage populations for downstream analyses. **(F)** AML patients were stratified according to overall survival, with 5 years used as the threshold. Patients surviving longer than 5 years were classified as the good-prognosis group, whereas those surviving less than 5 years were classified as the poor-prognosis group (top panel). The relative mRNA expression levels of FTO, ALKBH5, and ALKBH3 in macrophages isolated from the two groups were measured by RT-qPCR (bottom panels). Data are presented as the mean ± SD, and statistical significance was determined using unpaired t-tests (P < 0.05). * P < 0.05; *** P < 0.001.

Four m^6^A-regulated macrophage subsets modulate macrophage cell states, with eraser-high macrophages exhibiting pro-inflammatory characteristics, and reader-high as well as m^6^A-deficient subsets particularly involved in driving immunosuppressive programs. We therefore hypothesized that these macrophage states are closely linked to patient prognosis. To investigate the clinical relevance of macrophage m^6^A states, we analyzed three independent RNA-seq datasets from AML patients. We first applied AUCell to score the activity of m^6^A regulators in each patient (**Figure 6A**). The m^6^A abundance inferred from RNA-seq partially represents the m^6^A abundance of macrophages. In all cohorts, the m^6^A-deficient and reader-high populations were consistently dominant, suggesting a TME landscape skewed toward exhaustion and immunosuppression. To evaluate whether the m^6^A scores were associated with clinical and molecular characteristics, we examined the relationships between the three m^6^A scores and key clinical variables, including age, sex, FAB subtypes, and genetic alterations (28). We found that none of the three m^6^A scores showed a strong or consistent association with patient sex or age groups. Similarly, pairwise comparisons within groups revealed no significant differences in m^6^A scores across FAB subtypes or genetic alterations, with no clear enrichment of any specific category (**Supplementary Figure 4A**). Overall, these results indicate that the m^6^A-related scores are largely independent of conventional clinical features and known genetic alterations, supporting their potential role as independent prognostic indicators in AML.

We next examined whether these macrophage states correlated with patient prognosis. Kaplan-Meier survival analysis revealed that in both the GSE106291 and GSE146173 cohorts, elevated eraser activity, which may indicate a higher abundance of eraser-high macrophages, was associated with the most favorable survival outcomes (**Figures 6B, C**). In contrast, both reader-high and m^6^A-deficient scores, indicating a higher abundance of the corresponding m^6^A-regulated macrophages, were associated with poorer survival outcomes, with the latter predicting the worst prognosis. The writer-high score group was excluded from subsequent analyses owing to limited sample size and lack of statistical power. We constructed time-dependent ROC curves to evaluate the predictive capacity of m^6^A-regulated macrophage surrogate signature. The model demonstrated moderate predictive accuracy, as confirmed by the AUC curve analysis (**Figure 6D**). In GSE106291, the model showed relatively high specificity and PPV, particularly at longer follow-up times, indicating robust identification of high-risk patients. In contrast, in GSE146173, the model exhibited higher sensitivity and NPV, especially at 1 year, suggesting improved ability to exclude patients with favorable outcomes (**Supplementary Table 5**).

We performed a comparative analysis of the performance of two established models (Model1 (29): Risk score = 0.5890×UCP2 + 0.2590×DOCK1 -0.2193×SLC14A1 + 0.2553×SLC25A1, Model2 (30): ALKBH5 (coefficient= 0.0348), HNRNPA2B1 (coefficient=-0.0054), YTHDF3(coefficient=-0.0219), and METTL14 (coefficient=-0.0024). By calculating the concordance index (C-index) for our eraser-high prognostic model with favorable prognosis and the two previously published models, we found that our model demonstrated good discriminative ability, performing comparably to or even better than the existing models (**Supplementary Figure 4B**).

To validate the reliability of our findings, we collected clinical samples from AML patients and conducted long-term follow-up to record their prognostic outcomes. First, macrophages were isolated from patient samples using flow cytometry (**Figure 6E**). Subsequently, we examined the mRNA expression levels of three erasers (FTO, ALKBH5 and ALKBH3) in AML patient-derived macrophages. Based on a five-year survival threshold, patients were classified into good-prognosis and poor-prognosis groups. The results revealed that patients who survived for more than five years exhibited significantly higher expression levels of all three molecules compared to those with poor prognosis (**Figure 6F**). These observations from clinical samples were consistent with our bioinformatic analyses, supporting the notion that eraser-high macrophages are associated with a favorable prognosis in AML. Collectively, these findings suggest that patient-level m^6^A activity, reflecting the corresponding m^6^A-regulated macrophage states, may serve as a prognostic marker in AML.

## Discussion

m^6^A modification constitutes a key layer of epitranscriptomic regulation in cancer biology. As a key writer, METTL3 has been demonstrated to promote disease progression in multiple solid tumors, including bladder (31), gastric (32), and colorectal (33) cancers. Other writers also play critical roles, with METTL14 contributing to breast cancer (34) progression and WTAP promoting osteosarcoma (35). Among the erasers, FTO has been extensively studied (36) and is recognized as an oncogenic factor in cervical (37) and colorectal (38) cancers, while ALKBH5 (39) similarly facilitates colorectal cancer development. Within the reader family, the YTHDF proteins (YTHDF1 (40, 41), YTHDF2 (42, 43), and YTHDF3 (44)) have also been shown to exert important roles in cancer progression. m^6^A regulators are well-studied in solid tumors and leukemic cells, but their roles in shaping the AML immune microenvironment, especially macrophage phenotypes, remain underexplored. In this study, by systematically analyzing publicly available scRNA-seq datasets from AML patients, we identified four distinct m^6^A-regulated macrophage states with unique functional profiles: writer-high, a pro-proliferative state; eraser-high, a pro-inflammatory and antigen-presenting state; reader-high, an immunosuppressive state; and m^6^A-deficient, a functionally exhausted state. We demonstrate that m^6^A modifications govern macrophage functional reprogramming and shape the AML immune microenvironment.

Our study uniquely demonstrates that m^6^A modifications can influence macrophage functional reprogramming. Specifically, writer-high macrophages exhibit pro-proliferative changes, eraser-high macrophages display enhanced inflammatory and antigen-presenting functions, reader-high macrophages adopt immunosuppressive features, and m^6^A-deficient macrophages progress toward functional exhaustion. Notably, these patterns do not fully align with observations reported in solid tumors. For instance, YTHDF2 promotes tumor-supporting macrophage polarization in triple-negative breast cancer (TNBC) (45), WTAP facilitates M2 polarization and enhances pancreatic adenocarcinoma (PAAD) growth and metastasis (46), and ALKBH5 drives the formation of senescent foam macrophages in atherosclerosis (13). These differences may be due to these discrepancies arise for two main reasons. First, most prior studies focused on the effects of individual m^6^A regulators, whereas our analysis, based on a large cohort of 129 AML patients, captures the coordinated activity of multiple regulators at the single-cell level, providing a more comprehensive view of macrophage reprogramming. Secondly, differences between hematologic malignancies and solid tumors, including the composition of the tumor microenvironment and modes of immune dysregulation, may result in distinct effects of aberrant m^6^A expression in AML compared with solid tumors.

We investigated m^6^A-dependent inter-macrophage communication patterns and found that writer-high and eraser-high cells primarily transmit MIF–(CD74+CD44) signals to activate neighboring macrophages, whereas reader-high and m^6^A-deficient cells strongly promote macrophage proliferation through CD99-CD99 interactions, thereby contributing to an immunosuppressive microenvironment. Studies have shown that macrophages engage in intercellular communication with multiple cell types (47), influencing tumor cell states through interactions with cancer cells (48), while also interacting with immune cells such as CD4+ T cells to modulate the immune microenvironment (49). Our results further suggest that macrophages interact with T cells and other immune cells to modulate their activation states and functional properties. But research on macrophage–macrophage communication remains limited. Here, we show that distinct m^6^A states regulate neighboring macrophages through specific signaling, driving coordinated reprogramming. Single-cell transcriptomics reveal writer-high cells dominating early stages, eraser-high promoting M1 activation, reader-high favoring M2 polarization, and m^6^A-deficient cells inducing exhaustion. Unlike previous studies emphasizing developmental trajectories or subset heterogeneity (50), our work integrates m^6^A states to define their impact on macrophage differentiation and fate in AML.

An unfavorable prognosis continues to be a major challenge for patients with AML. Clinically, both newly diagnosed and relapsed AML patients have been reported to exhibit increased infiltration of M2-like macrophages, which is associated with poor prognosis (51). Mechanistically, M2 macrophages promote AML progression through multiple pathways, including direct mitochondrial transfer to leukemic blasts (52), secretion of CCL20 to maintain AML cells iron homeostasis and inhibit ferroptosis (6), facilitation of IL-34-driven accelerated disease progression (53). Macrophage-based immune checkpoint targeting, particularly anti-CD47 therapies, has been explored. The anti-CD47 antibody showed modest clinical responses when used as monotherapy in AML patients (54). Despite progress, AML immunotherapies remain constrained by safety and efficacy, highlighting the urgent need to further investigate the molecular mechanisms of immune evasion in leukemic cells to identify more effective therapeutic targets (55). Rigorous dissection of RNA modification marks and regulators in tumor cells and immune cells is considered as a fundamental and crucial for developing effective interventions (56). Our study revealed that macrophage states enriched in the eraser-high subpopulation are associated with favorable prognostic outcomes, a finding further validated using macrophages isolated from AML patient samples. This observation suggests that differential expression of m^6^A regulatory molecules within macrophages may serve as a potential biomarker for prognostic stratification in AML patients. Therefore, m^6^A-based targeted strategies may provide new avenues for prognostic stratification and therapeutic intervention in AML.

Several limitations should be noted. First, due to data unavailability, comparison with the ELN 2022 risk stratification system could not be performed. Second, m^6^A modification levels were not directly measured because of limitations in data type and sample availability. Future studies integrating well-annotated cohorts and direct m^6^A profiling will be required to address these limitations.

## Conclusion

Our study presents a single-cell transcriptomic atlas of AML macrophages, uncovering the role of m^6^A regulation in shaping their fate and function. Writers promote activation, erasers sustain inflammation, readers facilitate immune evasion, and m^6^A-deficient cells drive exhaustion. These regulators orchestrate downstream networks and signaling pathways, thereby reprogramming macrophage states. Higher abundances of Reader-high and m^6^A-deficient macrophages were associated with poorer survival, highlighting their impact on both immune modulation and clinical outcomes. Our findings highlight the regulatory role of the m^6^A system in LAMs and emphasize that targeting the epigenetic–immune crosstalk may represent a novel basis for prognostic stratification and a promising therapeutic strategy in AML.

## Data Availability

The datasets presented in this study can be found in online repositories. The names of the repository/repositories and accession number(s) can be found in the article/**Supplementary Material**.
